# Askin’s tumor: a case report and literature review

**DOI:** 10.1186/1477-7819-11-10

**Published:** 2013-01-22

**Authors:** Zineb Benbrahim, Samia Arifi, Khaoula Daoudi, Mounia Serraj, Bouchra Amara, Mohammed Chakib Benjelloun, Nawfel Mellas, Omar El Mesbahi

**Affiliations:** 1 Medical Oncology Department, Hassan II University Hospital, Fès, 30006, Morocco; 2 Service of Pneumology, Hassan II University Hospital, Fès, 30006, Morocco

## Abstract

Askin’s tumor is a primitive neuroectodermal tumor developing from the soft tissues of the chest wall. Its diagnosis approach is complex and requires a multidisciplinary team. Given the rarity of this entity, no regimen has been validated in the literature. We report two cases of Askin’s tumor with a major response to polychemotherapy and surgical resection in one case. These cases show that treatment of Askin’s tumor should be multimodal, requiring discussion in multidisciplinary tumor working groups.

## Background

Ewing sarcoma and peripheral primitive neuroectodermal tumor (previously called peripheral neuroepithelioma) were originally described in the early 1900s as distinct clinicopathologic entities. They are rare malignant small round cell tumors that arise from the primitive nerve cells of the nervous system, but they can also occur outside the central nervous system in the chest wall, pelvis and extremities. Askin’s tumor is a primitive neuroectodermal tumor (PNET) of the thoracopulmonary region described the first time in 1979 by Askin *et al*. about 20 white children and adolescents [1]. It develops from the soft tissues of the chest wall, particularly in the paravertebral region. Askin’s tumor occurs in young Caucasian adults and is associated with poor prognosis. Given the rarity of this recent disease, no regimen has been validated in the literature. We report two cases of Askin’s tumor with a major response to polychemotherapy and review different data about diagnosis and treatment of this entity.

## Cases presentation

### Case 1

**Figure 1:**
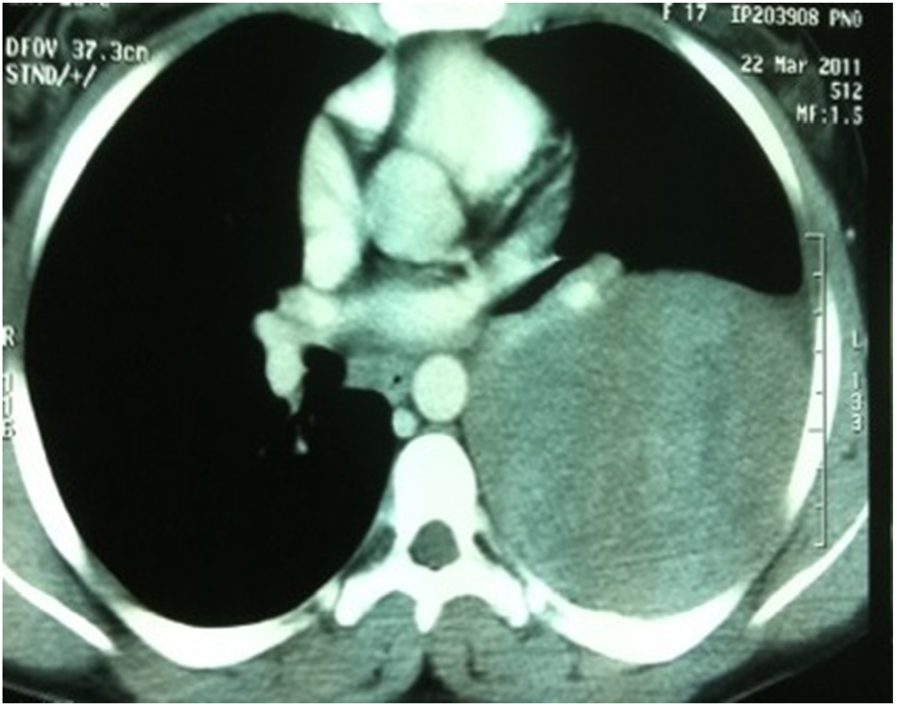
Large mass on the right lower rib cage developing from the outside of parenchyma.

**Figure 2:**
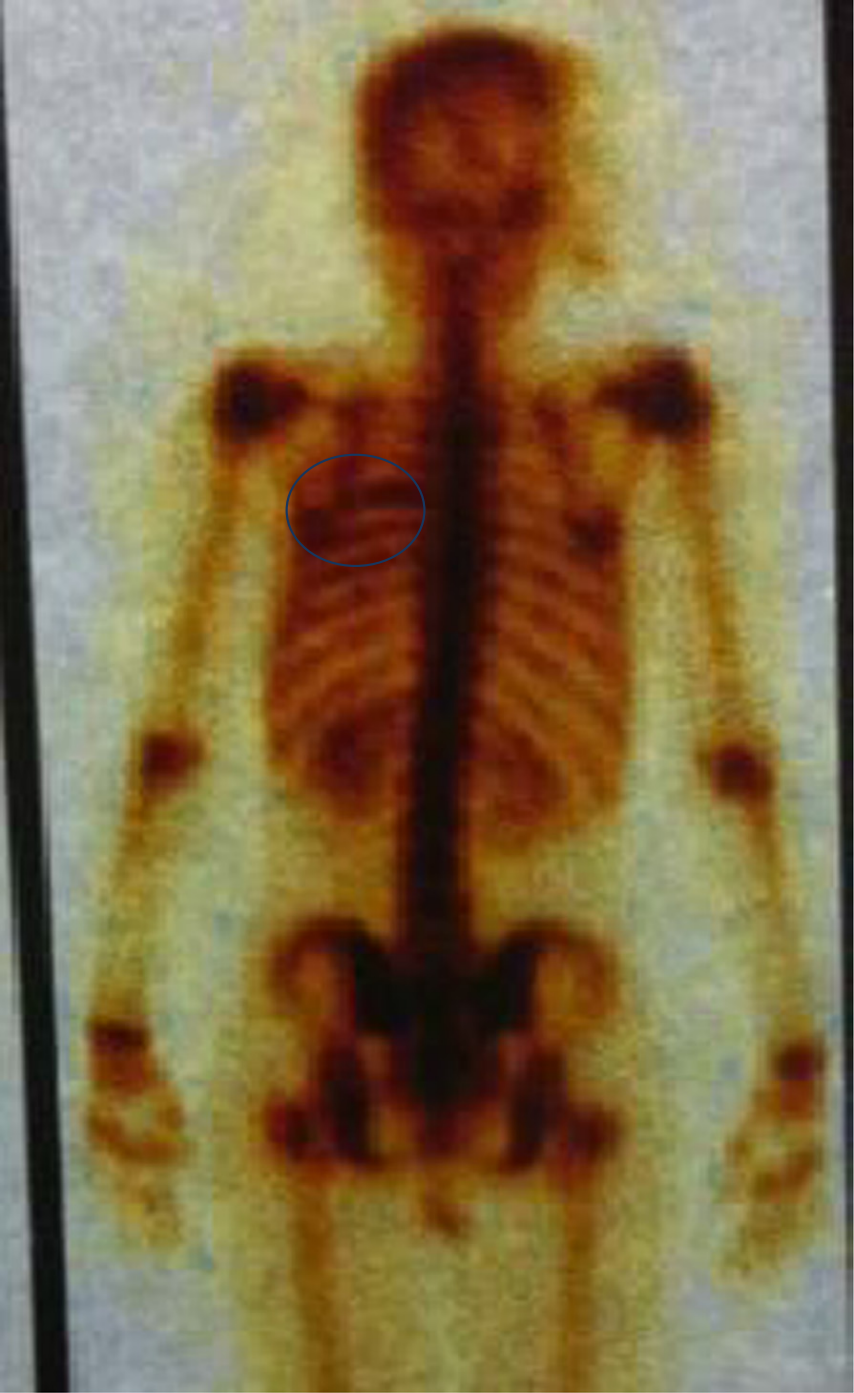
Bone scan showing a hyperfixation of the sixth rib.

A 17-year-old female presented two months’ history of cough, weight loss and pain in the right side of the chest. Physical examination revealed decreased breath sound in the right lung without any other abnormality. Laboratory investigations were normal. A chest radiograph showed an opacity in the right hemithorax with partial destruction of the sixth rib. To well document this opacity, a computed tomography (CT) scan was justified. The CT scan revealed a large lobulated mass on the right lower rib cage developing from the outside of parenchyma with erosion of the sixth rib (Figure 1). A CT-guided biopsy showed a malignant, lobular tumor with characteristic Homer Wright rosettes. A diagnosis of PNET of the chest wall (Askin Rosai tumor) was made. An isotope bone scan confirmed that no other skeletal site was involved (Figure 2). The tumor was classified as T1N0M0. The patient received four cycles of induction chemotherapy including ifosfamide, vincristine, and adriamycin. She then reported marked improvement in chest pain. A repeat CT scan showed disappearance of the chest tumor with residual erosion of the sixth rib. She was a candidate for surgical resection. However, she refused the intervention. To date, she remains disease-free after 10 months of follow-up.

### Case 2

A 27-year-old female was admitted for cough, dyspnea, pain in the right chest and weight loss. Physical examination showed a 5 × 5 cm rounded fixed mass on the right chest wall. Laboratory examinations were within normal limits. A chest radiograph and chest CT scan showed a soft-tissue density mass in the right rib cage with partial destruction of the fifth and sixth ribs and pleural effusion. No mediastinal or axillary lymphadenopathy was found. Pleural biopsy revealed a small cell tumor which stained negative for glycogen but immunocytochemically positive for neuron-specific enolase (NSE) and CD99. T (11;22) research has not been carried out. The diagnosis of Askin’s tumor was made. An abdominal CT scan did not show any metastatic localization. An isotope bone scan confirmed that no other skeletal site was involved. The tumor was classified as T2N0M0. After four cycles of chemotherapy of doxorubicin and cyclophosphamide, we observed a major clinical and radiological regression of the soft tissue mass. The primary tumor was resected, including the fifth and sixth ribs. Histology revealed residual microscopic tumor compatible with primitive neuroectodermal tumor. The patient received six courses of adjuvant chemotherapy. To date, she remains disease-free, with no evidence of recurrence after three years of follow-up.

## Discussion

Ewing sarcoma (ES) and peripheral primitive neuroectodermal tumor (PNET) were originally described as distinct clinicopathologic entities: in 1918, Stout described a tumor of the ulnar nerve composed of small round cells focally arranged as rosettes; this entity was subsequently designated neuroepithelioma, and then PNET [2]. Later, in 1921, ES was described as an undifferentiated tumor involving the diaphysis of long bones. It was also reported to arise in soft tissue (extraosseous ES) [3]. Nevertheless, it has become clear, over the last three decades, that these entities comprise the same spectrum of neoplastic diseases known as the Ewing sarcoma family of tumors (EFT), which also includes malignant small cell tumor of the chest wall and atypical ES. ES of the chest wall were originally reported by Askin *et al*. in 1979 in 20 white children and adolescents (average age, 14 years) [1]. Since then, PNETs within the thoracopulmonary region have been termed Askin tumors. This is a rare disease in the pediatric group, and even rarer in adults. Caucasian ethnic group is clearly predominant [4]. It is presented as a painful wall mass, often associated with dyspnea, cough, weight loss, Horner’s syndrome, or regional lymphadenopathy. A chest wall soft-tissue density mass, sometimes associated with rib erosion and/or pleural effusion, is the commonest radiographic manifestation [5]. The most important role of the CT scan is to confirm the presence of a solid chest wall and to demonstrate their possible intrathoracic extension and/or direct lung invasion [5,6]. Magnetic resonance imaging (MRI) findings of Askin tumors have been described as heterogeneous soft-tissue masses with a moderate to high signal intensity on T1-weighted images, greater than skeletal muscle, and high signal intensity on T2-weighted images [5,6].

Histological examination shows an undifferentiated sarcomatous tissue small round cell with scant eosinophilic cytoplasm, high nuclear/cytoplasmic ratio, small single nucleoli, and a high mitotic rate. Morphologically, these features are similar to that of other small round cell tumors involving bone and soft tissue, including lymphoma, small cell osteosarcoma, mesenchymal chondrosarcoma, dedifferentiated synovial sarcoma, desmoplastic small round cell tumors, rhabdomyosarcoma and medulloblastoma. As a group, these tumors cause difficult diagnostic problems when examined by light microscopy alone. Although no routinely used immunohistochemical stain can positively distinguish EFT from other undifferentiated tumors, the majority of EFT expresses high levels of a cell surface glycoprotein (designated CD99, MIC2 surface antigen or p30/32MIC2) that is encoded by the CD99 (MIC2X) gene. Another new potential immunohistochemical marker for EFT is NKX2.2, the protein product of the NKX2-2 gene [7].

Cytogenetic or molecular genetic studies looking for particular chromosomal translocations t (11; 22) (q24; q12) and/or their fusion transcripts are usually required to secure the diagnosis [5]. However, it raises the question of its relationship with ES, which presents a similar translocation. Marina *et al*. suggested the following criteria to retain the diagnostic of Askin’s tumor [8,9]:

• development of the tumor from a peripheral nerve;

• identification of Homer-Wright rosettes;

• expression of NSE and Leu-7;

• existence of cytoplasmic neurosecretory granules and microtubules

• identification of t (11; 22) (q24; q12);

• detection of proto-oncogenes (N-myc, C-myb, C-ets-1);

• activity of neurotransmitter biosynthetic enzymes (tyrosine hydroxylase, dopamine B hydroxylase and acetylcholine transferase).

Given the rarity and the recent individualization of this disease, no regimen has been validated in the literature. In contrast to extraosseous osteogenic sarcomas when management follows the principles established for soft-tissue tumors rather than primary bone tumors, ES is treated in the same multidisciplinary manner regardless of whether it arises in bone or soft tissue. Treatment includes radical surgical resection, neoadjuvant and adjuvant chemotherapy and radiation [10]. Most clinical centers performing intensive chemotherapy are reporting long-term survival rates between 60 and 70%, suggesting that ES is sensitive to anticancer agents. Current protocols of chemotherapy are based on a combination of two to six anticancer drugs, which are continued after locoregional treatment for up to twelve months [11]. These drugs include doxorubicin, actinomycin D, cyclophosphamide, ifosfamide, vincristine, etoposide, busulfan, melphalan and carboplatin. The most classic associations are: VACA (vincristine, doxorubicin, cyclophosphamide, actinomycin), VAIA (replacing cyclophosphamide with ifosfamide), EVAIA (addition of etoposide to VAIA) and finally VIDE (vincristine, ifosfamide, doxorubicin, etoposide) [12].

Used radiotherapy doses vary between 50 and 60 Gy. The prognosis of Askin’s tumor is very poor. Askin reported that 14 of 18 patients with known prognosis died four to forty-four months after diagnosis, and the mean survival period was eight months [13]. Local recurrences are very common. Furthermore, metastases are already present at diagnosis in 10% of cases [14].

## Conclusion

In summary, we experienced two cases of Askin’s tumor with a major response to polychemotherapy. These cases show that treatment of Askin’s tumor should be multimodal. Discussion in multidisciplinary tumor working groups is indeed warranted.

## Consent

Written informed consents were obtained from the patients for publication of this case report. A copy of the written consent is available for review by the journal’s Editor-in-Chief.

## Competing interests

The authors declare that they have no competing interests.

## Authors’ contributions

The work presented here was carried out in collaboration between all authors. ZB, KD and SA analyzed the data and wrote the manuscript. MS, BA and MCB have been involved in the acquisition of clinical data. NM and OE participated in reviewing the scientific literature and contributed to the final version of the manuscript. All authors read and approved the final manuscript.
